# Words and arbitrary actions in early object categorization: weak evidence for a word advantage

**DOI:** 10.1098/rsos.230648

**Published:** 2024-02-21

**Authors:** Ricarda Bothe, Léonie Trouillet, Birgit Elsner, Nivedita Mani

**Affiliations:** ^1^ Psychology of Language, Georg-August University Goettingen, Gottingen, Niedersachsen, Germany; ^2^ Leibniz Science Campus 'Primate Cognition', Goettingen, Germany; ^3^ Developmental Psychology, University of Potsdam, Potsdam, Germany

**Keywords:** word learning, action learning, novelty preference, visual object categorization, infancy

## Abstract

Both words and gestures have been shown to influence object categorization, often even overriding perceptual similarities to cue category membership. However, gestures are often meaningful to infants while words are arbitrarily related to an object they refer to, more similar to arbitrary actions that can be performed on objects. In this study, we examine how words and arbitrary actions shape category formation. Across three conditions (word cue, action cue, word–action cue), we presented infants (*N* = 90) with eight videos of single-category objects which vary in colour and other perceptual features. The objects were either accompanied by a word and/or an action that is being performed on the object. Infants in the word and action condition showed a decrease in looking over the course of the familiarization phase indicating habituation to the category, but infants in the word–action condition did not. At test, infants saw a novel object of the just-learned category and a novel object from another category side-by-side on the screen. There was some evidence for an advantage for words in shaping early object categorization, although we note that this was not robust across analyses.

## 1. Introduction

Words are accorded a special role in early development. Unlike other auditory cues, such as tones, words lead infants to focus on the commonalities among objects and, thereby, facilitate category formation (e.g. [1–5]). In the visual domain, other cues, such as non-verbal gestures and motions, have similarly been shown to facilitate categorization (e.g. [2,6]). However, gestures are linked in meaningful ways to the objects on which they are performed or to which they refer. Words, in contrast, are arbitrarily linked to the objects to which they refer. Can seemingly arbitrary actions associated with objects influence the formation of categories in a way akin to the role of words? Arbitrary actions that can be performed on objects are more comparable to the arbitrariness of word–object associations than goal-directed gestures or motions. Against this background, the current study will investigate whether the well-documented effect that words facilitate category formation can be replicated using other arbitrary cues in the input, such as arbitrary actions. In what follows, we first outline the literature on how words influence category formation, followed by a brief review of the literature on how other cues in the input, like gestures or motions, influence early object categorization.

Words have been shown to influence visual category formation from very early on in development. For example, Ferry and colleagues [7] showed that when a label—but not a tone sequence—consistently accompanied objects from a particular category, even 3-month-olds show evidence of having formed a basic object category (e.g. all dinosaurs). Such findings are typically explained by suggesting that labels direct the infant's attention to perceptual commonalities among objects that share category membership [1,4,7–10]. Words may also go beyond merely triggering sensitivity to similarities in visual input during category learning, but may also introduce concepts that are not perceptually apparent onto a visual scene (e.g. [10–12]). For example, Fulkerson & Haaf [3] reported that, by 9 months old, infants form very basic object categories (e.g. horses, airplanes) when objects were accompanied by labels, sounds and in silence (e.g. no additional cue to visual object). However, when infants were presented with objects from more global, superordinate categories (e.g. animals, vehicles), only infants in the label condition showed category learning at test, suggesting that labels may be particularly powerful in shaping category formation, especially in the absence of category-related perceptual cues. Results from such studies have, therefore, been taken to suggest that language influences concept activation and category formation differently from non-linguistic information [2–4,13,14].

At the same time, some studies report no differences in the extent to which words and sounds impact early categorization success. For example, Robinson & Sloutsky [15] familiarized 8- and 12-month-olds with a single-object category in silence or paired with either a word or sound and found increased attention to the objects during familiarization when they were accompanied by words and sounds, relative to when they were presented in silence. However, increased attention to the objects during familiarization in the word and sound condition was not mirrored in infants' category formation at test (see also [9,15–18]). In the former high-powered study (*N* = 162), infants showed improved category formation in the silent condition relative to when the objects were accompanied by words or sounds, suggesting that auditory cues impaired, rather than boosted category formation. Thus, while language influences infants’ attention to objects, Robinson & Sloutsky [15] find no evidence of an advantage for language in shaping early categorization.

Differences in the extent to which infants attend to word–object mappings relative to sound–object mappings may be driven by the familiarity of such cues. For example, words are more familiar to young infants relative to non-linguistic stimuli [9,15], because infants are exposed to language from early on. Thus, language may preferentially shape categorization due to infants' increased familiarity with linguistic cues. If infants’ increased attention to objects when accompanied by linguistic cues stems from their increased familiarity with language, then other familiar communicative cues should modulate infants' attention, and potentially categorization, to a similar extent.

Actions and gestures are frequent and extremely salient cues in the infant's immediate social environment. For instance, when caregivers interact with their infants, they often exaggerate and repeat movements with their hands [19] and use infant-directed actions such as pointing, giving, and showing [20,21] to increase infants' attention towards visual stimuli and the overall communicative input [22,23]. By their first birthdays, infants actively encode goal-directed actions or movements, i.e. actions that are performed on an object that results in an event or effect, to structure their visual input. For example, 11-month-olds showed evidence of categorizing objects with limited perceptual overlap (i.e. a single critical part was perceptually similar across objects), when objects were introduced with a goal-directed action during familiarization (i.e. placing the object on an apparatus allowed the experimenter to pull out a coloured band), but not in absence of such cues [24]. However, such cues had no influence on infants’ categorization of objects with increased perceptual overlap. Such findings suggest that object–action associations, just like object labels (cf. [3]), can guide category formation when objects share limited perceptual overlap.

Studies directly comparing the influence of linguistic versus goal-directed action cues in visual categorization suggest that the latter may be more salient in shaping the formation of object categories. For instance, Booth & Waxman [2] familiarized 14- and 18-month-olds with objects sharing perceptual overlap accompanied by either a word cue, an action cue (e.g. jiggle or slide) or no cue. At test, infants saw a novel member of the familiarized category next to a novel object from an unseen category and were asked to touch/point to the object from the familiarized category, i.e. at test, infants were presented with the objects in the presence of the goal-directed action or word cue. Both age groups identified the target object at test in the goal-directed action cue condition. However, only 18-month-olds, but not 14-month-olds, identified the target object upon hearing its label, suggesting that goal-directed action cues—but not word cues—may trigger generalization of a novel instance of a familiar category early in development.

A more recent study by Sučević *et al*. [6] investigated the effect of words and motions in visual category learning. Here, 10-month-olds were given the opportunity to explore objects from two novel categories that were accompanied by either a word, a motion (i.e. the objects moved up and down or side to side of their own volition) or no cue in a gaze-contingent design. Here too, infants showed improved category formation when the objects were accompanied by motions relative to words. Critically, category formation in this task was assessed by comparing infants' looking behaviour to a novel object (here a composite of the two categories) relative to a prototype of the two trained categories, in the absence of additional word or motion cues. Additionally, gaze-contingent exploration showed that infants focused more on perceptually overlapping features across objects when they were presented with a cue (word or motion) relative to the no cue condition, especially when the objects were accompanied by motion cues. Thus, motion-, and potentially, word cues may drive infants’ attention to the perceptual overlap between objects, thereby triggering generalization of objects with shared object features, leading infants to sort these objects into a common category (at least when accompanied by motion cues).

Results from such studies suggest that both word- and visual-dynamic cues support infants' encoding and recognition of category-specific properties. There is a crucial difference, however, in the association between the object and goal-directed action cues and object motions, on the one hand, and words, on the other hand. Goal-directed action cues, for instance, provide additional information about causal form–function relations. Object motions, similarly, are initiated by the object and are related to the affordances of the object features. Words, on the other hand, are arbitrarily related to objects and their features and may only cue visual categories once they have been successfully associated with object features in the first few trials. A more comparable examination of how infants use visual and linguistic information to cue category membership is afforded by examining the role of words and arbitrary actions in curing category membership.

Against this background, the current study examines how presenting arbitrary actions and words together with members of novel object categories cues infants’ categorization of the novel objects. This would allow us to examine how naturally occurring communicative cues in the infants' environment shape how infants categorize objects in the visual world. Across three conditions, we examined the extent to which infants categorize perceptually similar objects into a single category when objects were accompanied by either novel word cues, novel arbitrary actions being performed on the objects or both word and arbitrary action cues. We included the latter condition in order to examine whether there was a word boost in category formation when infants were presented with both word and action cues in synchrony alongside the novel object category members. If category learning were equally influenced by accompanying words and actions, then infants should show similar evidence for category formation in both the word cue and the action cue condition. However, given that actions and gestures precede the emergence of words in early development (e.g. [20,25]), there may be an action-bias in category formation at around 1 year of age. Of particular interest is the extent to which the presentation of simultaneous word and action cues shapes category formation. On the one hand, words and actions often co-occur in the input with some accounts suggesting that the temporal alignment of words and actions in the input may boost learning of word–object associations [26,27]. If multisensory redundancy were to similarly impact category formation, we would expect a boost in category formation in the word–action cue condition relative to the word cue and action cue condition. On the other hand, the simultaneous presentation of words and actions may detract attention from the perceptual similarities of the objects presented, potentially hindering category formation in the word–action cue condition.

Category formation is typically investigated by presenting infants with a series of perceptually overlapping objects from a single visual category during familiarization and then testing infants on their looking behaviour to a novel member of the familiarized category and a member of a different as-yet novel category [1,5,28]. Longer looking times to the novel object from an unknown category relative to the novel object from the familiarized category at test are typically interpreted as generalization of the objects from the familiarized category and index object category formation [29] (see also [30]). Some studies also examine infants’ habituation to objects from the category during familiarization, with a decrease in looking time towards same-category objects over the course of the familiarization phase being interpreted as infants' habituation to the category [1,5,31]. Our assessment of category formation included both the proportion of looking time to the novel member of the familiar category relative to the novel member of the unknown category at test as well as the decrease in looking time to same-category objects during familiarization as an index of habituation to the category. In particular, we were interested in the extent to which arbitrary actions and words impact infants’ object category formation, independently and in combination.

## 2. Material and methods

### 2.1. Participants

Infants were aged 1 year (*N* = 90, females = 47, *M*age = 11.02 months, *SD*age = 1.07 months, age range = 10–14 months) and grew up in a German-monolingual environment (i.e. infants were exposed to German for greater than 80% [32]). Infants were born at term or less than two weeks before term and parents reported no auditory or visual deficits with regard to their child's development and provided data for at least 50% of the training phase (i.e. look at the screen for at least half of a training trial, for four trials) as well as for the test phase (i.e. look at the screen for at least half of a test trial, for a single trial). Participation was rewarded with a book. The ethics committee of the Georg-Müller Institute for Psychology in Goettingen provided ethics approval for this project (number 276).

We pre-registered a sequential Bayesian analysis, where we computed a sequential Bayes factor (SBF) [33,34] following collection of data from individual children to examine the likelihood of the data under the alternative hypothesis, H_1_ (infants look longer to the object from the familiar as opposed to the novel category), relative to the null hypothesis, H_0_ (there is no difference in looking to the object from the familiar as opposed to the novel category). Here, if the BF exceeds a pre-specified minimum level of evidence (1/3 < BF < 3; i.e. moderate evidence for either hypothesis) for the alternative or null hypothesis, we would stop collecting data from additional children. We started computing the SBF following collection of data from a pre-specified initial sample of 20 children, in order to ensure that the initial sample size justified our belief in the effect [33,34]. We planned, therefore, for our final sample size to be determined by the SBF values. However, we stopped data collection at 90 infants in order to optimize resource allocation.

### 2.2. Materials

**Figure 1.**
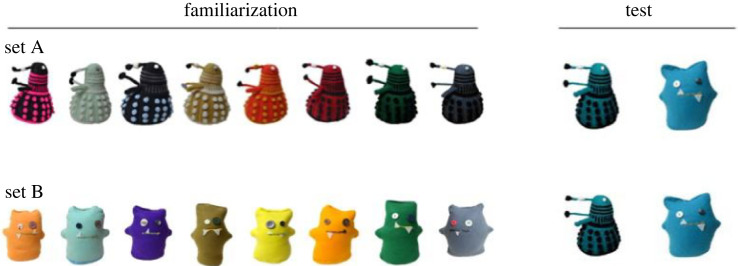
Objects used in the training and test phase. Note. Infants were familiarized with eight objects from a single category (i.e. either set A or set B and then tested on their novelty preference as we presented them with one object from the just-familiarized category and one object from a never-seen-before category.

We designed and produced two categories of soft toys to present infants with novel objects which were 20 cm tall and 9 cm wide, figure 1.

The final object sample (18 out of 24 soft toys) was chosen based on a visual similarity study with adults which took place online (*N* = 35, female = 20). Adults were presented with colour photographs (12 soft toys of group A and 12 soft toys of group B) and asked to rate the similarity of object pairs within and across category items using a Likert-type scale from 1 (very dissimilar) to 6 (very similar). Similarity was rated via button press (1–6) on a computer keyboard. Pictures of object pairs and a similarity scale were shown on screen until participants pressed a button. Participants were provided with no auditory information during this study. Out of the 12 objects of category A and 12 objects of category B, we chose the nine objects in each category with the highest similarity scores relative to the objects in the other category. Objects included in the final sample for the infant study were all rated above 4 (out of 6), ranging from 4.17 to 5.03 in category A and from 4.14 to 5 in category B. We aggregated participants' ratings on object similarity within a category (i.e. each soft toy from category A with each other soft toy from category A) and between categories (i.e. each soft toy from set A with each soft toy from set B). Across participants, within-category ratings for soft toys from both categories were similar (set A: *M* = 4.44, *SD* = 1.11; set B: *M* = 4.43, *SD* = 1.12) and there was no difference in ratings between the two categories, *t*_11.99_ = −0.06, *p* = 0.95. Similarity ratings were primarily driven by object shape (see [35] for a shape bias in similarity detection), i.e. ratings of colour-matched toys between categories were different from the within category ratings, *t* = 2.41, *SE* = 1.14, *p* = 0.017.

In total, 24 videos of the objects were created to form familiarization sets of eight stimuli in each category across each of the three conditions. In the *word cue condition*, infants saw the object standing still on a table centred to the screen, with the agent's right hand on the table by the side of the object, palm facing down. The auditory stimuli for the training phase consisted of single presentations of the label, i.e. ‘Oh, ein Tanu!’ (Oh, a Tanu!), and were roughly 2 s long. Infants heard a label (i.e. ‘Tanu’ or ‘Loeki’, which are in keeping with German phonotactic constraints) produced by a female German-native speaker in infant-directed speech twice at 1000 ms and 6000 ms after video onset.

**Figure 2.**
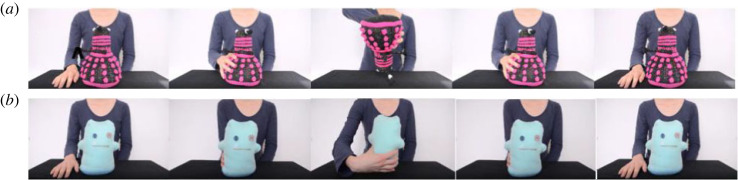
Visualizations of actions performed on the soft toys. Note. Infants saw eight objects from a single category either being turned upside down and back up again (*a*) or being rotated along its axis (*b*).

In the *action cue condition*, infants see the agent's hand performing an arbitrary action on each object and were provided with no auditory cue, figure 2. Within each video, action–object presentations were shown twice for each object. Videos began with the object standing in the middle of the screen and the agent's right hand to the right side of the object, palm placed down on the table. 1000 ms after video onset the agent's hand starts to grasp the object and, across videos, either turns the object upside down and back up again or rotates the object along its axis, with the hand again by the side of the object facing down at the end of the video. The action is then repeated once more in the video, 2000 ms after the completion of the first action (i.e. 6000 ms after video onset). Each individual action was roughly 3 s long.

In the *word-action cue condition*, infants see the action being performed on each object while simultaneously hearing the label (i.e. ‘Tanu’ or ‘Loeki’) in the presence of each object in a synchronized manner, i.e. onset of word–object and action–object presentations was temporally aligned [27]. Timing of presentation of stimuli was identical to the word cues and action cues described above.

At test, infants were presented with a photograph of an as yet unfamiliar object that overlapped perceptually with the objects presented during training alongside an image of an unfamiliar object that did not overlap perceptually with the objects presented during training, i.e. from the other category. Objects presented in the videos in the familiarization phase varied in colour, while objects presented side-by-side at test matched in colour.

### 2.3. Design

Infants participated in a novelty-preference task that consisted of a training phase and a subsequent test phase (i.e. individual training and test trials were 10 s each). Infants were randomly assigned to one of the three conditions which differed with regard to the cue that was presented with the object during the familiarization phase only (word cue, action cue, word–action cue). Order of stimulus presentation during training and test was randomized for each participant. The labels and actions that co-occurred with specific categories were counterbalanced across infants, meaning that each infant saw one single action and one single label for all objects presented to them. Thus, one group of infants heard the word ‘Tanu’ or saw the upside-down action with objects from category A, while another group of infants heard the word ‘Tanu’ or saw the upside-down action with objects from category B. The same was true for the other label and action.

#### 2.3.1. Training phase

The training phase consisted of 8 trials. In each trial, infants saw a video of a novel object from a single category. Object presentation was counterbalanced across participants such that half of the children saw objects from set A during training and the other half saw objects from set B.

#### 2.3.2. Test phase

In the test phase, infants saw photographs (854 × 480 pxl) of two objects side-by-side on the screen. Both trials were identical apart from left/right side presentation of test objects and lasted 10 s each. Test objects were novel objects from set A and set B, such that the infant saw a photograph of an object sharing perceptual overlap with objects presented during training and one object from the other unfamiliar category. Infants were provided with no auditory stimuli during these windows. Longer looking times to the object from the unfamiliar category are typically interpreted as evidence for novelty-preference and taken to suggest that infants successfully categorized the objects presented during training.

### 2.4. Procedure

Infants sat either in a baby seat or on their caregiver's lap at a distance of 60–55 cm away from a display (92 cm × 50 cm) in a dimly lit experimental room. A remote eye tracker (Tobii X 120) placed underneath the display recorded gaze data at 60 Hz and Tobii Pro Lab software was used for stimulus presentation. Two loudspeakers placed above the display presented auditory stimuli, while visual stimuli were presented on the display. Two cameras located immediately above the display provided online recordings of the infant and were used to keep track of the infant during the course of the experiment. We used a five-point grid calibration (a dot appearing in every corner and one in the centre of the screen) in Tobii Pro Lab. The experiment was initiated following successful calibration and the first trial started when infants were fixating the screen.

## 3. Results

Raw data files, analysis scripts, analysis output files and plots can be accessed via OSF (see [36] and https://osf.io/a4rdt and https://osf.io/emd3p for manuscript pre-registrations). In the following, we present the data and results separately for the training and test phases. Overall, we excluded data from additional infants due to eye-tracker calibration issues (*n* = 7), infant movement during the experimental procedure (*n* = 1), bilingualism (*n* = 2) and parental interference at test (*n* = 1).

### 3.1. Training data

Altogether, the final sample provided us with 713 training trials (99.1% of all trials shown during the familiarization phase) while the average infant provided looking time data for 7.92 out of 8 possible training trials. During the familiarization phase, we examined whether there were differences in the amount of time infants spent looking at the screen when the objects were presented with accompanying words (word cue condition), actions (action cue condition) or words and actions (word–action cue condition). This allows us to identify potential differences in the extent to which infants habituated to the object category, i.e. showed a decrease in looking towards the objects with increasing number of trials, when they saw objects accompanied by a word, action, or word–action cue. The response was total looking time towards the object on screen in each trial.

To test the effect of *condition* on infant's looking during familiarization, we conducted a full-null model comparison. The full model was a generalized linear mixed effects model (GLMM) [37], using the function *lmer* of the package lme4 (version 1.1-23) [38]. The between-participant factor *condition* in interaction with the covariate *trial number* entered the fixed effect structure of the full model. In order to ease likelihood of model convergence and to enhance interpretability of estimate of coefficients, we z-transformed the covariate *trial number*. To allow for infant specific variation in looking behaviour across the training trials for the individual infant, we add *infant ID* as a random intercepts effect and *trial number* on infant ID as a random slope (i.e. to minimize type I errors).

**Figure 3.**
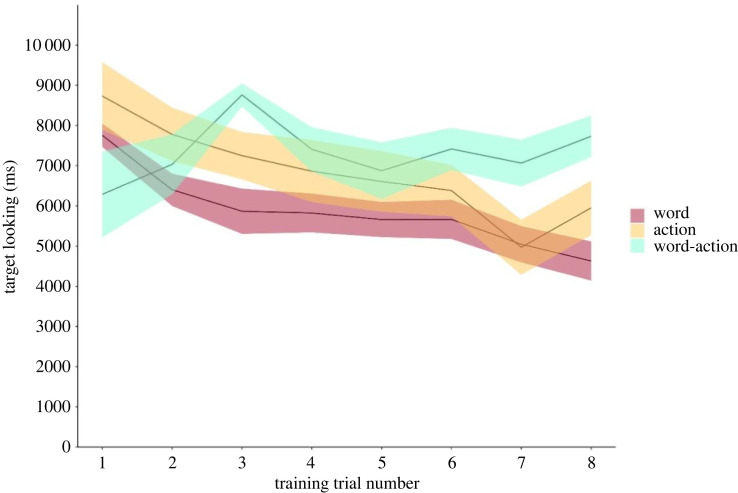
Infant's looking trajectory towards objects during familiarization.

To avoid cryptic multiple testing [39], we compared this full model with the null model lacking *condition* and *trial number* as well as their interaction in the fixed effects parts but being identical to the full model in the random effects part. This comparison was significant, $\chi_{\left(4,N=90\right)}^{2}=41.46$, *p* < 0.001. We then used drop1 analyses to identify which part of the fixed effect structure contributed to changes in the response, which revealed that infants' looking times were modulated by an interaction between *condition* and *trial number*, *F*_2,88.81_ = 7.24, *p* < 0.001 (table 1). In other words, the extent to which infants decreased their looking towards object on screen over the course of familiarization phase, i.e. habituated to the presented category, differed across conditions, figure 3. As indicated by the significant interaction noted above, figure 3 suggests that infants showed a decrease in their looking times towards the familiarization objects across trials in the word cue and action cue conditions, but not in the word–action cue condition.

**Table 1.** GLMM estimating the effect of condition, trial number and their interaction on infants' target looking during object category familiarization.

predictors	estimate	min.	max.	std error	lower CI	upper CI	statistic	*p*-value
**(intercept)**	8.594	8.573	8.639	0.066	8.465	8.719	131.003	**<0.001**
**condition [action]**	0.447	0.402	0.491	0.093	0.276	0.608	4.818	**<0**.**001**
**condition [word-action]**	0.504	0.459	0.525	0.093	0.313	0.675	5.435	**<0**.**001**
**trial.number**	−0.168	−0.18	−0.148	0.039	−0.248	−0.093	−4.272	**<0**.**001**
**condition[action]:trial.number**	−0.012	−0.033	0.012	0.056	−0.122	0.098	−0.218	0.827
**condition[word-action]:trial.number**	0.177	0.144	0.189	0.056	0.07	0.288	3.181	**0**.**002**

Note. The overall interaction of *condition* with *trial number* significantly impacted infants' looking trajectory to the training objects (*F* = 7.24, *p* < 0.001).

While the decrease in looking time to the object on screen over trials did not differ between the action and the word condition, *t*_88.79_ = −0.218, *p* = 0.827, there was a significant difference in the extent to which infants habituated to the objects presented on screen across word and word–action trials, *t*_88.84_ = 3.181, *p* = 0.002. However, despite the absence of a difference in the extent to which infants habituated in the word and action condition, infants spent longer overall looking at the object on screen in the action, *t*_90.03_ = 4.818, *p* < 0.001, and the word–action condition, *t*_90.223_ = 5.434, *p* < 0.001, relative to the word condition. We estimated model stability by omitting individuals individually, fitting the full model to each of the derived subsets, and then comparing the subset estimates to the estimates for the full data sets. This revealed the model being of good stability (see again table 1 for minimum and maximum estimates).

### 3.2. Test data

Infants provided us with a total of 175 test trials from 180 test trials shown (97.2%), with 85 infants delivering data for both test trials and 5 infants delivering data for a single test trial (word = 59 trials, action = 59 trials, word–action = 57 trials). In what follows, we first examine the extent to which infants’ looking behaviour, in particular, the proportion of time they spent looking at the unfamiliar object from the familiarized category relative to the object from the novel category, was modulated by the cues that accompanied presentation of these objects during familiarization, i.e. in the word cue, action cue and word–action cue condition.

We first report the results of our preregistered sequential Bayesian analyses comparing the proportion of target looking to chance in a Bayesian *t*-test separately for each condition. As reported above, we collected an initial sample of 20 children in each condition and then sequentially collected data from children in each condition and computed a sequential Bayes factor upon adding individual children in each condition. The SBF in each condition is reported in table 2. As table 2 suggests, the SBF in the action and the word–action condition already crossed the threshold for the H_0_ at 20 children (action cue condition, SBF = 0.306 and word–action cue condition, SBF = 0.276), while in the word cue condition, the SBF crossed the threshold for H_0_ at 21 children. We, however, continued testing children in all conditions to guard against a false negative [33]. In the word–action condition, we stopped data collection at 30 children after we consistently found evidence for H_0_ (SBF at 30 children = 0.198). SBFs for the action condition varied between 0.247 and 0.419. In other words, SBFs never neared the threshold for H_1_ and consistently suggested anecdotal and, at 30 children, moderate evidence for H_0_ in the action cue condition (SBF = 0.281). Thus, in both action and word–action conditions, we find consistent evidence for H_0_ being true, given the data, i.e. that there was no significant difference in looking to the object from the familiar as opposed to the novel category.

**Table 2.** Development of the Bayes factor for each child per condition when *n* ≥ 20.

condition	SBF scale	BF development based on *n*										
		20	21	22	23	24	25	26	27	28	29	30
action	medium	0.306	0.247	0.262	0.320	0.419	0.350	0.333	0.336	0.338	0.360	0.281
	wide	0.229	0.182	0.194	0.239	0.318	0.263	0.249	0.252	0.253	0.270	0.208
word	medium	0.336	0.304	0.407	0.331	0.409	0.533	0.460	0.620	0.868	0.931	1.091
	wide	0.253	0.227	0.309	0.248	0.310	0.409	0.350	0.479	0.680	0.731	0.860
word–action	medium	0.276	0.249	0.237	0.233	0.243	0.219	0.210	0.213	0.206	0.203	0.198
	wide	0.205	0.184	0.174	0.171	0.179	0.160	0.153	0.155	0.150	0.147	0.144

**Figure 4.**
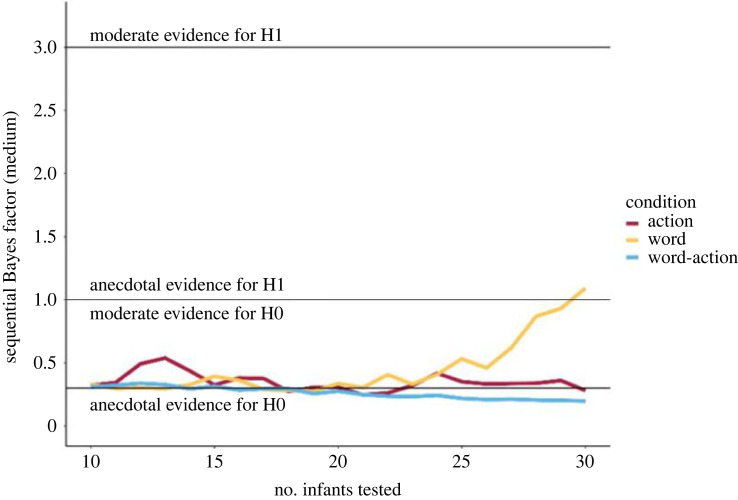
Sequential Bayes factor development with increasing sample size per condition. Note. We present a sequential Bayes factor (SBF) plot demonstrating data across three conditions, with the number of infants tested increasing from 10 to 30 per condition. Data for datasets with fewer than 10 children are not included as the SBF lacks meaningful interpretability with such limited sample sizes.

In the word cue condition, while we found evidence for H_0_ during testing, this pattern changed considerably with additional infants. With 30 children, we find anecdotal evidence for H_1_ and stopped data collection. Thus, as far as the Bayesian analysis is concerned, our results are inconclusive with regards to whether we find a difference in looking to the object from the familiar as opposed to the novel category in the word condition (figure 4).

Next, we examine the effect of condition on infant's looking behaviour at test using a GLMM analysis [37]. Here, the response was the proportion of target looking (PTL, i.e. looks to target divided by looks to target and distractor in a single test trial). The target here was defined as the object from the novel category, and the distractor was the other object from the familiarized category. The model was fitted assuming a beta error distribution [40] and logit link function [41], and analyses were conducted in R (version 4.1.2 or higher [42]) using the functions ‘glmmTMB’ of the equally named package (version 1.1.1 or higher [43]).

Since the response included values being exactly 0 or exactly 1 (i.e. the infant spent either all of the trial looking at the target or all of the trial looking only at the distractor), we transformed the PTL response variable to allow for interval level assumptions of the beta distribution [44]. With regard to the predictors, *condition (word, action or word-action cue)*, the covariate test *trial number* (two levels: 1 and 2), as well as the difference between the looking time slope for each individual infant and the overall (fixed effect) slope during the familiarization phase entered the model as fixed effects. In order to increase likelihood of model convergence and to enhance interpretability of the estimate of coefficients, we z-transformed the covariates *trial number* at test and individual *habituation slope* during training. Since the dataset comprised only two observations per individual, the GLMM did not comprise any random slopes [45] but a random intercepts effect of *infant ID* and a random slope of *trial number* within infant ID.

**Figure 5.**
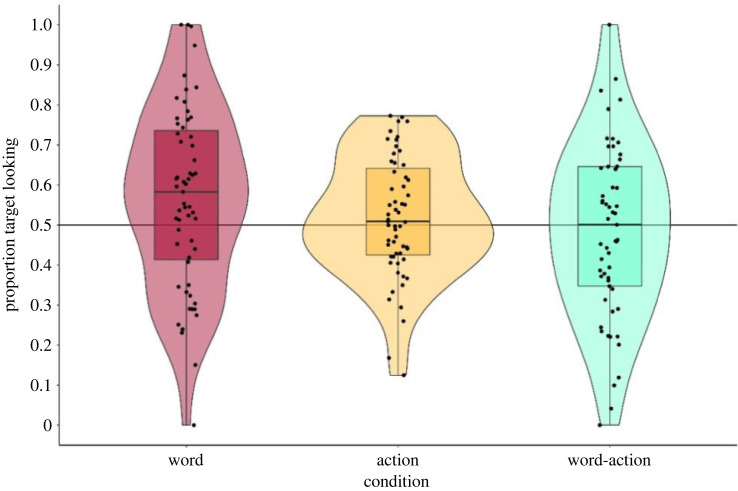
Infants’ proportion target looking at test per condition.

To examine the contribution of *condition* on infants' looking preferences at test, we compared this full model to its respective null model [39], with the null model lacking *condition* in the fixed effect structure but being identical with regard to the random effect structure. This comparison test was based on a likelihood ratio test [46] and was not statistically conclusive as to whether *condition* contributed to the variation in the response, $\chi_{\left(2,90\right)}^{2}=5.393$, *p* = 0.067. Drop1 analyses revealed that infants' *individual habituation slope* (*p* = 0.853) and test *trial number* (*p* = 0.785) did not account for variation within the response, nor did *condition* meet conventional threshold levels of statistical significance (*p* = 0.067). However, full model estimates revealed that proportion of target looking in the word condition (as reference level) differed from chance (*p* = 0.018), suggesting that infants in the word condition looked significantly more at the object from the novel category relative to the object from the familiar category at test, figure 5. The model was not overdispersed and of good stability, table 3.

**Table 3.** Word reference: infants' PTL modelled based on condition, trial number and habituation slope.

predictors	estimate	min.	max.	std error	lower CI	upper CI	statistics	*p*-value
**(intercept)**	0.316	0.266	0.358	0.133	0.051	0.577	2.366	**0.018**
**condition [action]**	−0.26	−0.3	−0.2	0.162	−0.553	0.064	−1.609	0.108
**condition [word-action]**	−0.374	−0.478	−0.303	0.163	−0.686	−0.065	−2.287	**0**.**022**
**habituation slope**	−0.013	−0.081	0.02	0.068	−0.138	0.12	0.034	0.853
**trial.no**	0.044	−0.045	0.123	0.16	−0.259	0.33	0.074	0.785

Note. Word data were set to the reference level of condition.

Similar to the training model results, looking preferences in the word cue condition did not differ from those derived from the action cue condition (*p* = 0.108), but did, indeed, differ from the word–action cue condition (*p* = 0.022), see again table 3.

While there was no statistical difference between the word and action cue estimates, we note that when the estimates were derived using the action cue condition as the reference level, the model indicated that the infants’ looking behaviour in the action cue group did not differ from chance (*p* = 0.681), see table 4. Thus, there was no significant difference in infants' looking behaviour to the object from the novel and familiar category when they saw the objects being moved in the familiarization phase. Neither was there a difference in looking behaviour in the action and the word–action condition (*p* = 0.49).

**Table 4.** Action reference: infants’ PTL modelled based on condition, trial number and habituation slope.

predictors	estimate	min.	max.	std error	lower CI	upper CI	statistic	*p*-value
**(intercept)**	0.056	0.026	0.101	0.136	−0.195	0.328	0.411	0.681
**condition [word]**	0.26	0.2	0.3	0.162	−0.063	0.577	1.609	0.108
**condition [word-action]**	−0.114	−0.211	−0.048	0.165	−0.43	0.168	−0.690	0.49
**habituation slope**	−0.013	−0.081	0.02	0.068	−0.139	0.117	0.034	0.853
**trial.no**	0.044	−0.045	0.123	0.16	−0.258	0.34	0.074	0.785

Note. Action data were set to the reference level of condition.

## 4. Discussion

In the above study, we examined how words and arbitrary actions individually and in combination impact infants’ categorization of perceptually overlapping objects. To examine this, we familiarized infants with different objects belonging to a single perceptual category, accompanied by either word, action or word–action cues. Categorization in such tasks is typically indexed by infants habituating to the category during familiarization, i.e. showing reduced looking towards novel tokens of the category as familiarization continues, or looking more towards a novel object from a perceptually distinct category relative to a novel object from the same category at test. In the current study, infants in the word and action condition, but not in the word–action condition, habituated to the novel object category during familiarization. As we argue below, this provides evidence of category formation to some extent in the word and action, but not in the word–action condition. When tested on their looking behaviour to novel objects from the familiarized and a novel category in the following test phase. Using a sequential Bayesian analysis, we found moderate evidence for the H_0_ in the action and word–action condition, i.e. that infants did not look longer to novel objects from the novel category relative to the familiarized category when objects were accompanied by either action or word–action cues during familiarization. We found anecdotal evidence for H_1_ in the word condition, i.e. anecdotal evidence that infants looked longer to the novel object from the novel category relative to the familiarized category at test when objects were accompanied by a word cue during familiarization. By contrast, a regression model suggested evidence for categorization in the word, but not in the action or word–action condition. Given that this pattern of findings was not robust across analyses, we call for caution in interpreting our findings below.

One reason for the differences across the analyses reported here is that disparities among infant data may appear less pronounced when employing Bayesian analyses as opposed to frequentist approaches [47]. In particular, testing more children may yield more power to the Bayesian analyses reported above, accumulating further evidence for the alternative hypothesis, akin to the regression analyses reported above. To the extent that this explanation accounts for the inconsistency in our results across analyses, it tentatively suggests a potential advantage for words in impacting early object categorization.

On the one hand, our tentative findings of a word advantage in early categorization are in keeping with previous work concluding that words support infants' categorization of objects (e.g. [5,11,12]). On the other hand, there is some debate regarding the extent to which these earlier findings capture the influence of the label in object categorization, especially when infants have not learned the association between the label and the object category [5,48–50]. In particular, these studies suggest that word effects in categorization may represent rudimentary levels of object categorization that do not involve learning of associations between labels and categories. Instead, they may represent a momentary snapshot of cognitive processing. For example, while 10-month-old infants show evidence of label-based object categorization, they do not show recognition of the previously exposed object category upon labelling [50]. Thus, the tentative word effect observed in our data may capture more rudimentary aspects of categorization that do not necessarily entail the learning of associations between categories and additional cues. Against this background, and highlighting the tentative evidence for a word effect in categorization found in the results reported above, we will next summarize attempts to explain the role of words in early object categorization.

### 4.1. The word effect in object categorization

The effects of accompanying word cues on object categorization are typically explained by suggesting that words increase sensitivity to the perceptual commonalities between objects and, therefore, promote the formation of a common category (e.g. [1–4,10,12]). This may be due, in part, to the fact that words are symbolic features and can be used to represent a wide range of objects and concepts [51].

A related account suggests that language cues (label-feedback hypothesis) may warp the perceptual space such that the accompanying labels may force objects closer in perceptual space, based on the statistical regularities of certain word–object pairings (e.g. [13,52,53]). After a couple of presentations of word–object pairings during familiarization in the current study, infants may find it easier to recognize other objects that share object features as belonging to the same visual object category. Thus, words may play a particularly important role in allowing infants to generalize from specific instances of objects to broader visual categories. For example, Lupyan & Bergen [53] illustrate with an example that it is harder to train a monkey than a human to climb a tree to collect coconuts, due to our ability to explain the task to humans using language. While a word can be used to refer to a single object, such as a coconut, this reference applies to all coconuts, although each coconut looks slightly different. So, the word *coconut* allows for irrelevant details of the individual coconut (like variation in size and colour) to be abstracted over and serves as a stable reference to all coconuts. Other cues, such as actions, for instance, are specific to the individual demonstration of the action, such that reaching for a large coconut requires a hand gesture that is different from reaching for a small coconut. Actions may, therefore, not generalize over incidental features of objects, like the size of a coconut, and may not warp the perceptual space to aid detection of perceptual similarity in the same way that words do [53,54].

Alternatively, the influence of words on early categorization has been explained by suggesting that infants may treat the word as an additional feature of the object, which in combination with other perceptual cues guides category formation [5,49,55]. Thus, according to this feature-based account, it is not the case that words highlight the commonalities across the objects. Rather, the word is, here, merely a consistent common cue that accompanies members of the same category leading infants to form categories of objects that share similar perceptual features, and enlist these categories in learning the meanings of words.

### 4.2. No evidence for categorization when objects were accompanied by arbitrary actions (and in combination with words)

While such an account would potentially predict a similar influence of other consistent accompanying cues on early categorization, we found no evidence for category formation at test when infants were presented with objects accompanied by actions or word–action cues. Indeed, the Bayesian analysis we report suggested moderate evidence that there was no difference in infants’ looking behaviour to novel objects from the familiarized and a novel category, when these objects were accompanied by action or word–action cues during familiarization. Similarly, the regression model found no evidence to suggest infants discriminated between these objects in their looking behaviour at test. This finding stands in contrast to previous studies suggesting an influence of functional movements [2] and object-initiated motions [6] on object categorization. In contrast to such cues that are more constrained by the affordances of the objects or are more intrinsic to the object (in the case of object-initiation motions), arbitrary actions are simply performed on the object and do not confer additional meaning to the action–object association. Thus, for example, infants could not relate an action goal to the action performance on objects within the category because arbitrary actions produced no visible or audible effects. Indeed, infants' expectation of an action effect or some kind of communicative intent, may have deterred attention to the perceptual features of the object and disrupted category formation. One explanation of these findings may, therefore, be that arbitrary actions do not have the same ability to reference meaning in a symbolic way (like words) and may therefore fail to cue category membership.

### 4.3. Words and actions influence infants’ habituation to objects from the same category

We did, however, find differences in the extent to which infants in the word, action and word–action cue conditions attended to objects during category familiarization. In particular, during the familiarization phase, infants in word and action groups showed a decrease in looking to the novel object category members over the course of familiarization. Such a pattern of looking is typically interpreted as an index of habituation to the visual object category, and consequently, is somewhat considered as evidence of visual category formation. Infants' looking behaviour during familiarization may, therefore, be taken to suggest that word and action cues similarly influence generalization of common object features, while the simultaneous presentation of word and action cues appears to disrupt such processes involved in category formation.

Importantly, we note that infants, in general, looked at objects longer when they were accompanied by an action cue compared to a word cue. Indeed, one possible explanation for the failure to find differences in looking behaviour to novel objects from the familiarized and the novel category at test in the action condition may lie in this increased attention to objects when they were accompanied by actions. In particular, the increased attention to the videos when actions were performed on the object relative to when the objects were merely accompanied by words may index the increased salience of actions on objects, detracting attention from the perceptual features of the objects presented. In what follows, we will discuss why infants may have attended longer to objects presented with an arbitrary action relative to when objects were presented with a word, and why such attentional differences to word and action stimuli may have affected category learning.

### 4.4. Attentional differences across modalities

In early development, dynamic hand movements, such as grasping an object, emerge as robust and salient features of infants’ perceptual environment. For instance, 5-month-olds discriminate and retain observed actions by others relative to discriminating static images of female faces [56,57]. Videos of objects being moved by an agent are, therefore, likely to be more intriguing to infants than videos of stationary objects accompanied by words. Such differences in attention to objects during familiarization have also been shown to impact infants' performance at test in visual object categorization tasks. For example, Robinson & Sloutsky [15] showed that infants’ attention to objects when they were presented in silence versus when accompanied by a tone or a word, influenced whether infants showed evidence for visual category formation at test. In particular, word–object and sound–object presentations increased infants' visual attention to objects compared to silent presentations of objects. However, while infants in the silent condition showed evidence for object categorization at test, this was not the case for infants in the word and tone conditions. Increased attention to objects accompanied by additional cues during object exploration may then affect infants’ looking behaviour at test and/or disrupt categorization during familiarization. There may, therefore, be a sweet spot for (examining) category learning that is mediated by attention to objects, such that either too little or too much attention to the objects within the category detracts from categorization performance.

A further possibility is that infants found it more difficult to visually track an object when it was moving than when the object was stationary. Therefore, longer looking times for moving objects may reflect the time needed by infants to encode a moving object. Moving objects may disrupt infants' visual encoding to such an extent that it detracts from the typically presented novelty preference in such tasks at test. Additionally, infants who saw objects being moved may have expected the objects to be in motion at test as well. In this case, their expectations about the object from the familiar category would be violated because thus far, infants saw objects with perceptual overlap consistently presented with an action being performed on objects. They might even have been surprised to see both objects as stationary visuals on the screen, because up until the testing phase, objects were presented in motion, leading infants to expect objects from both the novel and the familiarized category to be in motion. Such violations of infants’ expectations may have resulted in more balanced looking times to both objects, thereby preventing them from showing novelty preference for the target object at test.

Alternatively, arbitrary actions performed on the object may have enabled infants to explore perceptual aspects of objects in depth, allowing them to better encode object detail compared to when the object was presented stationary. Indeed, during the action performance, objects were dynamically moved in space and presented from different angles, which revealed more perceptual details about the objects relative to when they were presented still. While the still presentation of objects in the word condition may have allowed for structured examination of the objects' perceptual commonalities, increased perceptual information about moving objects may have disrupted detection of perceptual overlap between objects. This, in turn, may lead to difficulties generalizing and conceptualizing these common object features. At the same time, we note that infants in the action condition habituated to the familiarized category, suggesting that they were sensitive to the fact that objects accompanied by a consistent action shared perceptual overlap. So why did infants in the action condition not show a novelty preference at test?

### 4.5. Modality preferences influence novelty preference at test

Aside from differences in attention to objects that were named, stationary or in motion, it is possible that the nature of the task commonly employed for testing category formation—the novelty preference procedure—may have introduced a bias towards a word advantage in categorizing objects compared to actions. This is due to infants generally exhibiting preferences for visual and auditory stimuli that depend on the context in which these cues occur, i.e. whether it is in a familiar or novel setting. For example, Emberson *et al.* [58] showed that 8- to 10-month-olds differed in their preference for visual (e.g. smiling faces) and auditory (e.g. nonwords like *vot* or *meep*) stimuli, when the stimuli were novel or familiar. Specifically, infants in their study were first familiarized with six faces and six nonwords and then tested on their novelty preference for either another face, or another nonword, respectively. They found that infants displayed a novelty preference for auditory stimuli and familiarity preference for visual stimuli, i.e. they preferred familiar visual stimuli over a novel visual stimulus and a novel auditory stimulus over familiar auditory stimuli. Such cue-specific preferences may have biased infants in the current study towards more of a novelty preference in the word cue condition, and a familiarity preference in the action cue condition, due to differences in infants’ preference for visual and auditory stimuli. This would suggest that, while infants may have categorized the objects in both the word and the action cue conditions, infants may be more likely to show category formation in the word relative to the action cue condition, simply because category formation was indexed by novelty preference. The extent to which infants encoded objects during familiarization can thus depend on the modality of the stimulus infants become familiar with, on the one hand, and affect their novelty preference at test, on the other.

### 4.6. The role of words in combination with actions on object categorization

We did not find evidence for category formation at test when infants were familiarized with the objects and heard the label for this object and saw an action being performed on the object, similar to the action-only cue condition. However, unlike the word–action cue condition, infants in the action cue condition habituated to the object category during familiarization. The simultaneous presentation of words, actions and objects apparently led infants to pay close attention to the objects on screen with little to no drop in attention and, thus, no evidence of habituation to the familiar object category. Specifically, infants in the word–action cue condition looked at objects longer and more consistently than in the other two conditions, suggesting that encoding of the objects during familiarization may have been limited (see [58,59]). In other words, simultaneous presentation of words and actions may have thrown off infants' attentional patterns to the shared object features as they explored visual objects. Similar to the action cue condition, infants’ gaze behaviour may be less structured when they observe objects being moved (and named) than when they see them stationary and hear their labels.

An alternative explanation for the lack of an effect in the word–action cue condition is that infants faced challenges when encoding and processing objects in motion because visual objects are more difficult to track when in motion. Indeed, infants younger than 12 months of age are more likely to attend to the spatio-temporal features of an object relative to its perceptual features, suggesting that encoding changes to an object's location or orientation at the same time as its perceptual features may be challenging for young infants [60]. By contrast, recent research reveals no difference in infants' object encoding when presented synchronously with a word in motion or statically: 6-month-olds were equally likely to discriminate between an object and another object overlapping in some perceptual features, regardless of whether the object was originally presented in motion or statically [61]. However, from a linguistic perspective, labelling objects in motion may still have introduced competition between object or the motion as the referent of the label, resulting in reduced categorization in the word–action condition. By 12 months, infants exhibit conceptual understanding of a variety of verb meanings, including intentional (i.e. to get) or transactional (to give) actions [62], and only later, by 18 months, can they acquire novel verbs for verb categorization based on more sophisticated morphosyntactic cues (e.g. the penguin is pratching [63]). Consequently, the competition between word and action cues in the presence of a novel object could imply a conflict between the use of a noun and a verb, disrupting category formation in this condition.

Even though actions and words can work together to enhance communication and learning [26], multimodal presentations of temporally aligned word–action–object triads in the current study were detrimental to infants’ visual object category learning. Such results are in line with the literature showing that word–object learning is disrupted when words are accompanied by actions in early development [14,64,65]. For instance, Bothe and colleagues (under review) familiarized 1- and 2-year-olds with novel, arbitrary word–action–object triads. Here, only 2-year-olds showed recognition of word–object and action–object associations, and that only when the word–object–action triads were presented synchronously. One-year-olds, in contrast, showed no learning of either word–object or action–object associations when these stimuli were presented synchronously (or sequentially). This study, therefore, extends findings from the associative learning literature by showing that combined word–action–object presentations similarly disrupt object categorization.

## 5. Conclusion

In conclusion, we found limited evidence for visual object categorization when objects from a single perceptual category were accompanied by words, but not when they were accompanied by an action or a combination of a word and action. Importantly, there were some inconsistencies across analyses. Specifically, the sequential Bayesian analysis found anecdotal evidence for categorization in the word cue condition and moderate evidence for absence of categorization (as indexed by looking behaviour at test) in the action and word–action cue conditions. In contrast, the GLMM found evidence for categorization in the word cue condition but not in the action or word–action cue condition. The differences between the statistical approaches may result from the fact that categorization effects are relatively subtle in early development [48], which may not be accounted for in Bayesian analyses because there is no general way to represent uncertainty in the background knowledge of the data in this approach [47]. We also found that infants habituated to the objects during familiarization in the word and the action cue conditions, but not in the word–action cue condition. While this finding can provide an index of categorization in the former two conditions, we note that, in general, infants looked longer at objects during familiarization in the action and the word–action cue condition relative to the word cue condition. The latter suggests that object encoding during familiarization may have been influenced by the objects being presented in motion. Taken together, our findings suggest that words, relative to actions and a combination of words and actions, may support categorization to an extent distinct from other cues that are equally arbitrary and familiar, albeit potentially more salient.

## Data Availability

The supporting data and digital research materials for this paper can be accessed through https://osf.io/jc7kv/files/osfstorage. Additionally, relevant research materials such as statistical tools, protocols, and software used in this study can also be accessed through https://osf.io/jc7kv/files/osfstorage. We adhere to the open data policy of *Royal Society Open Science* and are committed to making our research data and materials freely accessible to the scientific community. For any further inquiries regarding data accessibility, contact Ricarda Bothe (Ricarda.bothe@uni-goettingen.de); or visit https://doi.org/10.17605/OSF.IO/JC7KV [36] for a reference to the data set and analyses.

## References

[1] Althaus N, Mareschal D. 2014 Labels direct infants' attention to commonalities during novel category learning. PLoS ONE **9**, e99670. (10.1371/journal.pone.0099670) 25014254 PMC4094422

[2] Booth AE, Waxman S. 2002 Object names and object functions serve as cues to categories for infants. Dev. Psychol. **38**, 948-957. (10.1037/0012-1649.38.6.948) 12428706

[3] Fulkerson AL, Haaf RA. 2003 The influence of labels, non-labeling sounds, and source of auditory input on 9- and 15-month-olds’ object categorization. Infancy **4**, 349-369. (10.1207/S15327078IN0403_03)

[4] Fulkerson AL, Waxman SR. 2007 Words (but not tones) facilitate object categorization: evidence from 6- and 12-month-olds. Cognition **105**, 218-228. (10.1016/j.cognition.2006.09.005) 17064677 PMC2099297

[5] Plunkett K, Hu J-F, Cohen LB. 2008 Labels can override perceptual categories in early infancy. Cognition **106**, 665-681. (10.1016/j.cognition.2007.04.003) 17512515

[6] Sučević J, Althaus N, Plunkett K. 2021 The role of labels and motions in infant category learning. J. Exp. Child Psychol. **205**, 105062. (10.1016/j.jecp.2020.105062) 33508654

[7] Ferry AL, Hespos SJ, Waxman SR. 2010 Categorization in 3- and 4-month-old infants: an advantage of words over tones. Child Dev. **81**, 472-479. (10.1111/j.1467-8624.2009.01408.x) 20438453 PMC2910389

[8] Balaban MT, Waxman SR. 1997 Do words facilitate object categorization in 9-month-old infants? J. Exp. Child Psychol. **64**, 3-26. (10.1006/jecp.1996.2332) 9126625

[9] Sloutsky V, Robinson C. 2008 The Role of words and sounds in infants' visual processing: from overshadowing to attentional tuning. Cognit. Sci. **32**, 342-365. (10.1080/03640210701863495) 21635339

[10] Waxman S, Markow D. 1995 Words as invitations to form categories: evidence from 12- to 13-month-old infants. Cognit. Psychol. **29**, 257-302. 8556847 10.1006/cogp.1995.1016

[11] Ferguson B, Waxman S. 2017 Linking language and categorization in infancy. J. Child Lang. **44**, 527-552. (10.1017/S0305000916000568) 27830633 PMC5484598

[12] Waxman SR, Braun I. 2005 Consistent (but not variable) names as invitations to form object categories: new evidence from 12-month-old infants. Cognition **95**, B59-B68. (10.1016/j.cognition.2004.09.003) 15788158

[13] Lupyan G, Thompson-Schill SL. 2012 The evocative power of words: activation of concepts by verbal and nonverbal means. J. Exp. Psychol. Gen. **141**, 170-186. (10.1037/a0024904) 21928923 PMC4124531

[14] Puccini D, Liszkowski U. 2012 15-Month-old infants fast map words but not representational gestures of multimodal labels. Front. Psychol. **3**, 101. (10.3389/fpsyg.2012.00101) 22493588 PMC3318184

[15] Robinson CW, Sloutsky VM. 2007 Linguistic labels and categorization in infancy: do labels facilitate or hinder? Infancy **11**, 233-253. (10.1111/j.1532-7078.2007.tb00225.x) 33412736

[16] Robinson CW, Sloutsky VM. 2004 Auditory dominance and its change in the course of development. Child Dev. **75**, 1387-1401. (10.1111/j.1467-8624.2004.00747.x) 15369521

[17] Robinson CW, Sloutsky VM. 2008 Effects of auditory input in individuation tasks. Dev. Sci. **11**, 869-881. (10.1111/j.1467-7687.2008.00751.x) 19046156

[18] Sloutsky VM, Napolitano AC. 2003 Is a picture worth a thousand words? Preference for auditory modality in young children. Child Dev. **74**, 822-833. (10.1111/1467-8624.00570) 12795392

[19] Koterba EA, Iverson JM. 2009 Investigating motionese: the effect of infant-directed action on infants’ attention and object exploration. Infant Behav. Dev. **32**, 437-444. (10.1016/j.infbeh.2009.07.003) 19674793

[20] Capirci A, Contaldo A, Caselli MC, Volterra V. 2005 From action to language through gesture: a longitudinal perspective. Gesture **5**, 77-155.

[21] Cheung RW, Hartley C, Monaghan P. 2021 Caregivers use gesture contingently to support word learning. Dev. Sci. **24**, e13098. (10.1111/desc.13098) 33550693

[22] Brand RJ, Baldwin DA, Ashburn LA. 2002 Evidence for ‘motionese’: modifications in mothers' infant-directed action. Dev. Sci. **5**, 72-83. (10.1111/1467-7687.00211)

[23] Brand R, Shallcross W. 2008 Infants prefer motionese to adult-directed action. Dev. Sci. **11**, 853-861. (10.1111/j.1467-7687.2008.00734.x) 19046154

[24] Träuble B, Pauen S. 2007 The role of functional information for infant categorization. Cognition **105**, 362-379. (10.1016/j.cognition.2006.10.003) 17129581

[25] Bates E, Bretherton I, Snyder L, Shore C, Volterra V. 1980 Vocal and gestural symbols at 13 months. Merrill-Palmer Quarterly of Behavior and Development **26**, 407-423.

[26] Gogate LJ, Bolzani LH, Betancourt EA. 2006 Attention to maternal multimodal naming by 6- to 8-month-old infants and learning of word-object relations. Infancy **9**, 259-288. (10.1207/s15327078in0903_1) 33412680

[27] Gogate LJ. 2010 Learning of syllable–object relations by preverbal infants: the role of temporal synchrony and syllable distinctiveness. J. Exp. Child Psychol. **105**, 178-197. (10.1016/j.jecp.2009.10.007) 20004909

[28] Younger B. 1985 The segregation of items into categories by ten-month-old infants. Child Dev. **56**, 1574-1583. 4075875

[29] Bomba PC, Siqueland ER. 1983 The nature and structure of infant form categories. J. Exp. Child Psychol. **35**, 294-328. (10.1016/0022-0965(83)90085-1)

[30] Quinn PC. 1987 The categorical representation of visual pattern information by young infants. Cognition **27**, 145-179. (10.1016/0010-0277(87)90017-5) 3691024

[31] Althaus N, Westermann G. 2016 Labels constructively shape object categories in 10-month-old infants. J. Exp. Child Psychol. **151**, 5-17. (10.1016/j.jecp.2015.11.013) 26778468

[32] Byers-Heinlein K, Bergmann C, Savalei V. 2021 Six solutions for more reliable infant research. *PsyArXiv*. (10.31234/osf.io/u37fy)

[33] Mani N, Schreiner MS, Brase J, Köhler K, Strassen K, Postin D, Schultze T. 2021 Sequential Bayes factor designs in developmental research: studies on early word learning. Dev. Sci. **24**, e13097. (10.1111/desc.13097) 33544976

[34] Schönbrodt FD, Wagenmakers E-J, Zehetleitner M, Perugini M. 2017 Sequential hypothesis testing with Bayes factors: efficiently testing mean differences. Psychol. Methods **22**, 322-339. (10.1037/met0000061) 26651986

[35] Landau B, Smith LB, Jones S. 1992 Syntactic context and the shape bias in children's and adults' lexical learning. J. Mem. Lang. **31**, 807-825. (10.1016/0749-596X(92)90040-5)

[36] Bothe R, Trouillet L, Elsner B, Mani N. 2023 What is special about words in early visual categorization? [Data set]. Georg-August University Göttingen. (10.17605/OSF.IO/JC7KV)

[37] Baayen RH, Davidson DJ, Bates DM. 2008 Mixed-effects modeling with crossed random effects for subjects and items. J. Mem. Lang. **59**, 390-412. (10.1016/j.jml.2007.12.005)

[38] Bates D, Mächler M, Bolker B, Walker S. 2015 Fitting linear mixed-effects models using lme4. J. Stat. Softw. **67**, 1-48. (10.18637/jss.v067.i01)

[39] Forstmeier W, Schielzeth H. 2011 Cryptic multiple hypotheses testing in linear models: overestimated effect sizes and the winner's curse. Behav. Ecol. Sociobiol. **65**, 47-55. (10.1007/s00265-010-1038-5) 21297852 PMC3015194

[40] Bolker B. 2008 Ecological models and data in R. Princeton, NJ: Princeton University Press.

[41] McCullagh P, Nelder J. 1989 Generalized linear models. London, UK: Chapman and Hall.

[42] R Core Team. 2022 R: a language and environment for statistical computing. Vienna, Austria: R Foundation for Statistical Computing. See https://www.R-project.org.

[43] Brooks M, Kristensen K, Van Benthem KJ, Magnusson A, Berg CW, Nielsen A, Skaug HJ, Machler M, Bolker BM. 2017 glmmTMB balances speed and flexibility among packages for zero-inflated generalized linear mixed modeling. The R Journal **9**, 378-400. (10.32614/RJ-2017-066)

[44] Smithson M, Verkuilen J. 2006 A better lemon squeezer? Maximum-likelihood regression with beta-distributed dependent variables. Psychol. Methods **11**, 54-71. (10.1037/1082-989X.11.1.54) 16594767

[45] Barr DJ. 2013 Random effects structure for testing interactions in linear mixed-effects models. Front. Psychol. **4**, 328. (10.3389/fpsyg.2013.00328) 23761778 PMC3672519

[46] Dobson AJ. 2002 An introduction to generalized linear models, 2nd edn. Boca Raton, FL: Chapman & Hall/CRC.

[47] Wang P. 2004 The limitation of Bayesianism. Artificial Intelligence **158**, 97-106. (10.1016/j.artint.2003.09.003)

[48] Oakes LM, Ribar RJ. 2005 A comparison of infants' categorization in paired and successive presentation familiarization tasks. Infancy **7**, 85-98. (10.1207/s15327078in0701_7) 33430540

[49] Gliozzi V, Mayor J, Hu J-F, Plunkett K. 2009 Labels as features (not names) for infant categorization: a neurocomputational approach. Cognit. Sci. **33**, 709-738. (10.1111/j.1551-6709.2009.01026.x) 21585482

[50] Hu JF. 2008 The impact of labelling on categorisation processes in infancy. PhD thesis, Department of Experimental Psychology, University of Oxford, UK.

[51] DeLoache JS. 2004 Becoming symbol-minded. Trends Cogn. Sci. **8**, 66-70. (10.1016/j.tics.2003.12.004) 15588810

[52] Lupyan G. 2012 What do words do? Toward a theory of language-augmented thought. Psychol. Learn. Motiv. **57**, 255-297. (10.1016/B978-0-12-394293-7.00007-8)

[53] Lupyan G, Bergen B. 2016 How language programs the mind. Topics Cogn. Sci. **8**, 408-424. (10.1111/tops.12155) 26184465

[54] Lupyan G, Rakison DH, McClelland JL. 2007 Language is not just for talking: redundant labels facilitate learning of novel categories. Psychol. Sci. **18**, 1077-1083. (10.1111/j.1467-9280.2007.02028.x) 18031415

[55] Althaus N, Gliozzi V, Mayor J, Plunkett K. 2020 Infant categorization as a dynamic process linked to memory. R. Soc. Open Sci. **7**, 200328. (10.1098/rsos.200328) 33204445 PMC7657915

[56] Bahrick LE, Gogate LJ, Ruiz I. 2002 Attention and memory for faces and actions in infancy: the salience of actions over faces in dynamic events. Child Dev. **73**, 1629-1643. (10.1111/1467-8624.00495) 12487483

[57] Bahrick LE, Newell LC. 2008 Infant discrimination of faces in naturalistic events: actions are more salient than faces. Dev. Psychol. **44**, 983-996. (10.1037/0012-1649.44.4.983) 18605829 PMC2738585

[58] Emberson LL, Misyak JB, Schwade JA, Christiansen MH, Goldstein MH. 2019 Comparing statistical learning across perceptual modalities in infancy: an investigation of underlying learning mechanism(s). Dev. Sci. **22**, e12847. (10.1111/desc.12847) 31077516 PMC7294581

[59] Hunter MA, Ames EW. 1988 A multifactor model of infant preferences for novel and familiar stimuli. Adv. Infancy Res. **5**, 69-95.

[60] Mareschal D, Johnson MH. 2003 The ‘what’ and ‘where’ of object representations in infancy. Cognition **88**, 259-276. (10.1016/S0010-0277(03)00039-8) 12804813

[61] Lany J, Aguero A, Thompson A. 2022 The temporal dynamics of labelling shape infant object recognition. Infant Behav. Dev. **67**, 101698. (10.1016/j.infbeh.2022.101698) 35279469

[62] Buresh JS, Woodward A, Brune CW. 2006 The roots of verbs in prelinguistic action knowledge. In Action meets word, 1st edn (eds KA Hirsh-Pasek, RM Golinkoff), pp. 208-227. New York, NY: Oxford University Press. (10.1093/acprof:oso/9780195170009.003.0009)

[63] He AX, Lidz J. 2017 Verb learning in 14- and 18-month-old English-learning infants. Lang. Learn. Dev. **13**, 335-356. (10.1080/15475441.2017.1285238)

[64] Bothe R, Eiteljoerge SFV, Trouillet L, Elsner B, Mani N. Submitted. Better in sync: temporal dynamics explain multisensory word-action-object learning in early development. Infancy. 10.1111/infa.12590 38520389

[65] Eiteljoerge SFV, Adam M, Elsner B, Mani N. 2019 Word-object and action-object association learning across early development. PLoS ONE **14**, e0220317. (10.1371/journal.pone.0220317) 31393901 PMC6687139

